# Supplementary material for: Cerebral blood flow predicts differential neurotransmitter activity
Source: Sci Rep. 2018 Mar 6;8:4074. doi: 10.1038/s41598-018-22444-0 (PMC5840131; doi:10.1038/s41598-018-22444-0)
Supplement: Supplementary file 1 — Supplement 1 [file 41598_2018_22444_MOESM1_ESM.docx]

**Supplement 1**

**Cerebral blood flow predicts differential neurotransmitter activity**

Juergen Dukart1, Štefan Holiga1, Christopher Chatham1, Peter Hawkins*^2^*, Anna Forsyth*^3^*, Rebecca McMillan*^3^*, Jim Myers*^8^*, Anne R Lingford-Hughes*^8^*, David J Nutt*^9^*, Emilio Merlo-Pich1, Celine Risterucci1, Lauren Boak1, Daniel Umbricht1, Scott Schobel1, Thomas Liu4, Mitul A Mehta*^2^*, Fernando O Zelaya^2^, Steve C Williams*^2^*, Gregory Brown*^5^*, Martin Paulus*^5^*, Garry D Honey1, Suresh Muthukumaraswamy*^3^*, Joerg Hipp1, Alessandro Bertolino1,6, Fabio Sambataro1,7

1 *F. Hoffmann-La Roche, pharma Research Early Development, Roche Innovation Centre Basel, Basel, Switzerland.*

*^2^ Department of Neuroimaging, Institute of Psychiatry, Psychology & Neuroscience, King's College London, London, United Kingdom*

*^3^ School of Pharmacy, Faculty of Medical and Health Sciences, The University of Auckland, Auckland, New Zealand*

*^4^ Center for Functional MRI, University of California San Diego, 9500 Gilman Drive MC 0677, La Jolla, CA 92093, United States; Departments of Radiology, Psychiatry and Bioengineering, University of California San Diego, 9500 Gilman Drive, La Jolla, CA 92093, United States.* *Department of Psychiatry*

*^5^ University of California, San Diego, La Jolla, USA Veterans Affairs San Diego Healthcare System, San Diego, USA*

*^6^ Institute Of Psychiatry, Department of Basic Medical Science, Neuroscience and Sense Organs, University of Bari 'Aldo Moro'*

*^7^ Department of Experimental and Clinical Medical Sciences (DISM), University of Udine, Udine, Italy*

*^8^ Centre for Neuropsychopharmacology, Imperial College London, London, United Kingdom*

*^9^Department of Medicine, Centre for Neuroscience, Imperial College London, London, UK*

**Supplementary Methods**

**Drug mechanisms of action**

Haloperidol is a typical antipsychotic primarily prescribed for treatment of schizophrenia, Tourette syndrome, mania and bipolar disorder with a presumed main but not exclusive mechanism of action as a dopamine antagonist. Risperidone and olanzapine are atypical antipsychotic that are commonly used in schizophrenia and bipolar disorder. Their main effect of action is presumably due to their antagonist effect on 5-HT 2a serotonin and D2 receptors. Methylphenidate is an indirect catecholamine agonist acting primarily through reuptake inhibition of dopamine and norepinephrine. It is mainly prescribed for Attention Deficit Hyperactivity Disorder. Escitalopram is an indirect selective serotonin agonist acting through reuptake inhibition on the serotonin transporter. It is primarily applied as an antidepressant. Ketamine and midazolam are established anesthetics. Ketamine acts primarily through its antagonistic effect on the N-Methyl-D-aspartate receptor (NMDA) but has also various other mechanisms of action. Midazolam is a positive allosteric modulator of Gamma-Amino Butyric acid (GABA) acting through binding at the benzodiazepine site.

**ASL acquisition and cerebral blood flow (CBF) computation**

A summary of acquisition parameters is provided in Table S1 and representative single subject data for each sequence are presented in Figure S6.

**Study 1 and 2**: All scans were conducted on a GE MR750 3Tesla scanner using a 12-channel head coil. ASL image data were acquired using a pseudo-continuous Arterial Spin Labelling sequence (PCASL) with a multi-shot, segmented 3D stack of axial spirals (8-arms) readout with a resultant spatial resolution of 2x2x3mm. Three control-label pairs were used to derive a perfusion weighted difference image (Dai et al., 2008). The labelling RF pulse had a duration of 1.5s and a post-labelling delay of 1.5s. The sequence included background suppression for optimum reduction of the static tissue signal. A proton density image was acquired in 48sec using the same acquisition parameters in order to compute the CBF map in standard physiological units (ml blood/100mg tissue/min).

**Study 3:** All scans were conducted on a General Electric Signa EXCITE 3.0 T short bore system. CBF was acquired with a FAIR QUIPSS II Arterial Spin Labeling protocol using an 8 channel receive-only head coil and a body coil for RF transmission. The FAIR QUIPSS II sequence used a spiral-out readout with 4 interleaves and the following parameters: TI1 600ms, TI2 1600ms, TR 2500 ms, TE 3 ms, 220 mm FOV and 64 x 64 matrix, 3.4375 mm x 3.4375 mm in-plane resolution, 20 x 5 mm axial slices.

**Study 4:** PCASL data were acquired using a Siemens Magnetom Skyra 3.0 T scanner using a 20 channel head coil. At each session, 7 pairs of perfusion-weighted and control scans were performed (single shot echo planar imaging (EPI) having 42 slices of 3 mm with an in-plane resolution of 3×3 mm2, field of view 192×192 mm; flip angle 90°). Echo time (TE) = 15.62 ms, labeling duration of 1800 ms and a post labeling delay of 1800 ms was used. A proton density image was acquired using the same acquisition parameters in order to compute CBF maps in standard physiological units (ml blood/100mg tissue/min). Quantitative CBF maps were computed as proposed for this sequence (Alsop et al., 2015).

**Study 5:**  PCASL data were acquired on a Philips Intera 3.0 T scanner using a 32 channel head coil. At each session, 60 pairs of perfusion-weighted and control scans were performed (single shot echo planar imaging (EPI) having 17 slices of 7 mm with an in-plane resolution of 3×3 mm2, field of view 240×240 mm; flip angle 90°). Sensitivity-encoded (SENSE) factor 2.5, echo time (TE) = 13.9 ms at a delay of 1600ms, slice time of 35 ms was used. A proton density image was acquired using the same acquisition parameters in order to compute CBF maps in standard physiological units (ml blood/100mg tissue/min). Quantitative CBF maps were computed as proposed for this sequence (Alsop et al., 2015).

**Pre-processing of CBF data**

Pre-processing of all CBF data was performed using the Statistical Parametric Mapping (SPM12) software package (Friston et al., 1994). CBF data were first co-registered to individual structural T1 images. Structural scans were segmented and normalized into the Montreal Neurological Institute (MNI) space using the SPM Segment function. Deformation parameters derived from this normalization were then applied to the co-registered CBF data to bring them into the MNI space. Regional CBF changes (∆CBF) defined as effect sizes (mean change divided by standard deviation of change) were extracted for the 41 Brodmann and subcortical regions described below (Figure S4).

**Receptor density profiles**

All receptor density maps were extracted from Figure 1 of the publication by Palomero-Gallagher et al. (Palomero-Gallagher et al., 2015) and provide estimates of respective receptor densities (low, intermediate, high) for Brodmann areas and also for some subcortical structures using a 3 point system (e.g. 1=low, 2=intermediate, 3=high). For some regions intermediate levels of receptor densities between those 3 levels were reported. Those were coded as 1.5 or 2.5. For Brodmann regions with sub-partitions an average receptor density score was computed. BA1 though reported in the review publication was not included for further analyses as it is not covered by the Brodmann imaging atlas provided with the MRIcron tool (Rorden and Brett, 2000). These receptor density maps (41 regions in total, Table S2) were used for all subsequent univariate analyses. As cerebellum was not covered by the review publication and also not fully covered by all ASL sequences it was not included in further analyses. Additionally, as for each receptor map for about 5% of regions the density was reported as unknown we aimed to reduce data loss for the multiple linear regression analyses requiring a full data matrix. For this an interpolated version of the receptor density table was created replacing the missing values by the mode of other densities for the corresponding receptor. Importantly, all of the above assumptions made for receptor density extraction if it all may only reduce the sensitivity to detect potential associations between receptor densities and CBF due to the introduced noise. However, they do not anyhow bias potential findings towards detection of potential false positive effects. They can be therefore considered as a conservative approximation of underlying receptor densities.

**Receptor density estimates from dopamine transporter single photon emission tomography**

Baseline DAT-SPECT data of 174 healthy elderly volunteers (mean age ± SD: 61±11 years, 109 male) were extracted from the Parkinson’s Progression Marker Initiative database (PPMI, www.ppmi-info.org/). Written informed consent was obtained from all subjects. The study was approved by Institutional Review Boards/Independent Ethics Committees. All image pre-processing steps were performed using the Statistical Parametric Mapping 12 software package (SPM12, www.fil.ion.ucl.ac.uk/spm) and Matlab R2013.b (MathWorks). DAT-SPECT data preprocessing comprised normalization to an average size DAT-SPECT template with subsequent normalization into MNI space, smoothing with a Gaussian kernel of 8 mm full-width at half maximum and scaling to the global image mean. A mean image was then computed from these preprocessed data. Regional DAT density estimates were extracted from this mean image for the 41 regions described above.

**Receptor density estimates from flumazenil positron emission tomography**

Dynamic [11C]flumazenil PET scans were acquired in a previous study with full arterial blood sampling for quantitative compartmental modelling (Myers et al., 2012). Arterial input functions were interpolated and corrected for metabolism and the ratio of plasma to whole blood. 4-D images were corrected for attenuation and scatter, and reconstructed into 20 frames using filtered backprojection. These were analyses at the voxel level using spectral analysis (Cunningham & Jones, 1993), with 100 logarithmically distributed orthogonal basis functions between 0.0008 and 1 s-1, to create parametric maps of total distribution volume (VT), with 2.09x2.09x2.42 mm resolution. These individual volume of distribution maps calculated as the summed integral of the peaks after spectral analysis were used as individual GABAa density estimates. Up from this point the pre-processing was identical to the one for CBF data comprising coregistration to structural scans and normalization to MNI space. A mean image of the 6 participants was then used to obtain the regional GABAa receptor density estimates for the 41 evaluated regions. The study was approved by an independent ethics committee.

**Testing for validity of parametric assumptions**

The extracted receptor density maps provide only a very granular estimation with respect to expression strength of the specific receptors. Therefore, we first aimed to understand if applying parametric assumptions when computing Pearson correlations with such discrete maps provides a valid null hypothesis for significance testing. For this we performed 1000 permutations correlating random permutations of the obtained receptor density maps (randomly permuting the obtained receptor density values to the different regions) with each of the obtained CBF change maps (overall 143000 permutations) (Figure S1). A visual plot of the obtained p-values indicates if the obtained p-values follow the expected uniform distribution or are skewed towards lower p-values. Further, the applied multiple linear regression models make the assumption of normally distributed residuals. We tested the validity of this assumption for the residuals obtained in the multiple linear regression analyses using Shapiro Wilk tests for normality.

**Supplementary Results**

**Testing for validity of parametric assumptions**

The p-value distribution obtained when correlating the randomly permuted receptor densities with CBF changes was highly consistent with the expected uniform distribution (Figure S1). This suggests the validity of p-values obtained in the correlational analysis with the correct receptor density maps. Shapiro Wilk tests for normality revealed that residuals obtained from multiple linear regressions with all compounds do not differ from a normal distribution (all p>.084) suggesting validity of the assumptions underlying the application of the multiple linear regression models in this study.

**Within modality correlations of CBF changes and receptor densities**

A weak median correlation of r=.14 was observed among the different neurotransmitter systems obtained from Palomero-Gallagher et al. (Palomero-Gallagher et al., 2015) suggesting a rather low co-localization of extracted receptor density profiles (Figure S2). The strongest positive correlation (r=.72;p<.001) was observed between α1 and 5-HT 1a receptor density maps and the strongest negative correlation (r=-.45;p=.006) between α2 and D2 receptor density maps. DAT density estimates as obtained using DAT-SPECT were significantly correlated with D1 (r=.57;p<.001), D2 (r=.56;p<.001) and α2 (r=-.40;p=.009) densities.

Similarly, we used correlational analyses to directly compare CBF changes obtained for the different compounds. A median correlation strength of r=.07 was observed in these analyses (Figure S3). The largest correlation (leasing aside the expected high correlation between low and high doses of risperidone) was observed between haloperidol and the high dose of risperidone (r=.94;p<.001), followed by haloperidol and olanzapine (r=.84;p<.001). The strongest negative correlation, albeit non-significant, was observed between escitalopram and the low dose of risperidone (r=-.21;p=.179).

**Multiple linear regression results without interpolation of receptor density maps**

Highly similar results as compared to the interpolated maps were obtained in the analyses excluding regions with missing data (no interpolation) for at least one receptor density map from multiple linear regression analyses. The obtained overall models showed a significant effect for all datasets except midazolam and the negative control test retest data: escitalopram (F(13,13)=4.2;p=.007), haloperidol (F(13,13)=3.2;p=.023), methylphenidate (F(13,13)=2.7;p=.040), olanzapine (F(13,13)=2.6;p=.048), low dose of risperidone (F(13,13)=4.6;p=.005), high dose of risperidone (F(13,13)=4.1;p=.008), ketamine (F(13,13)=3.0;p=.028), midazolam (F(13,13)=1.5;p=.249) and test retest data (F(13,13)=1.3;p=.313). Single significant or marginally significant regressors for the significant overall models were as following. For escitalopram these regressors were the intercept (t(13)=2.7;p=.018), GABAa (t(13)=1.8;p=.095), α2 (t(13)=2.0;p=.073) and 5-HT 1a (t(13)=-3.0;p=.010). For haloperidol significant or marginally significant were observed for D1 (t(13)=2.1;p=.061) and D2 (t(13)=2.2;p=.047). For methylphenidate, a significant or marginally significant effect was obtained for the intercept (t(13)=-2.5;p=.024), α2 (t(13)=1.9;p=.076), 5-HT 2 (t(13)=-3.0;p=.010) and D2 (t(13)=2.2;p=.051) regressors. AMPA (t(13)=-1.8;p=.094), Nicotinic α4β2 (t(13)=-2.2;p=.049), D1 (t(13)=1.8;p=.088) and D2 (t(13)=1.9;p=.079) showed a significant effect for olanzapine. Low dose of risperidone was associated with the intercept (t(13)=2.3;p=.036), α2 (t(13)=-2.0;p=.072), 5-HT 2 (t(13)=2.7;p=.017) and D1 (t(13)=3.2;p=.007) receptor densities. High dose of risperidone was linked to M1 (t(13)=-1.8;p=.095), D1 (t(13)=2.9;p=.012) and D2 (t(13)=2.1;p=.054) receptor density maps. Ketamine was associated with the intercept (t(13)=-1.9;p=.078), NMDA (t(13)=1.9;p=.080) and nicotinic a4b2 (t(13)=2.4;p=.032) receptor density. Despite the overall non-significant model, midazolam was associated with the intercept (t(13)=-2.6;p=.021), GABAa (t(13)=2.7;p=.018), M1 (t(13)=-2.0;p=.069), M2 (t(13)=-1.9;p=.080) and D2 (t(13)=1.9;p=.077) receptor densities.

**Test retest reliability results**

Excellent test-retest reliability was observed for the correlational profiles between CBF change and receptor density maps for each of the three compounds for which two ASL acquisitions were available within each session. More specifically, the within session ICCs were as following: ICC(low dose of risperidone)=0.97, ICC(high dose of risperidone)=0.99, ICC(olanzapine)=0.94, ICC(haloperidol)=0.96. The same analysis yielded excellent reliability when comparing receptor density mappings of high and low dose of risperidone (ICC=.95). Slightly lower but still excellent retest reliabilities were observed when comparing within session standardized regression coefficients obtained from the multiple linear regression models: ICC(low dose of risperidone)=0.94, ICC(high dose of risperidone)=0.96, ICC(olanzapine)=0.83, ICC(haloperidol)=0.97. Finally, the between session ICC for the regression coefficients obtained for high and low dose of risperidone was 0.89.

**Figure S1** Results of correlational analyses with randomly permuted receptor density maps

**
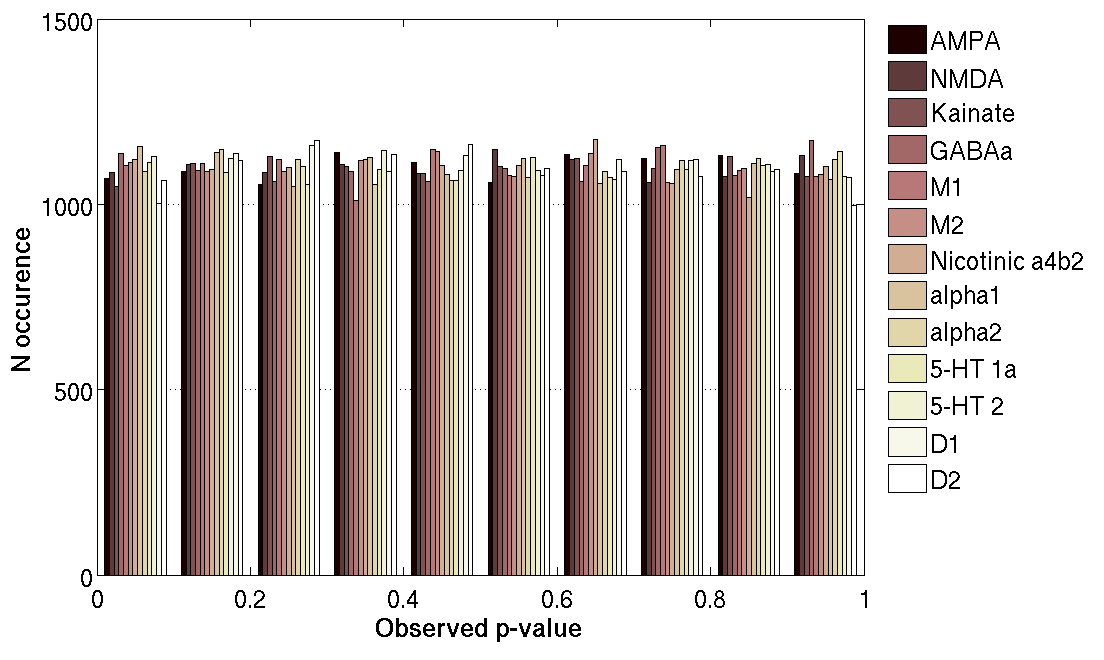
**

Number of p-values (N occurrence) obtained in correlations with randomly permuted receptor density maps is displayed for each p-value range (x-axis) and for each receptor density map (color scale)

**Figure S2** Results of correlational analyses between receptor density maps

 Pearson correlation coefficients and p-values (r;p) observed between respective receptor density maps are displayed. * dopamine transporter (DAT) density data were obtained from single photon emission computer tomography

**Figure S3** Results of correlational analyses between drug-induced CBF change maps

 Pearson correlation coefficients and p-values (r;p) observed between the corresponding CBF change maps for each drug are displayed

**Figure S4** Drug induced cerebral blood flow effect sizes for different cortical and subcortical regions

**
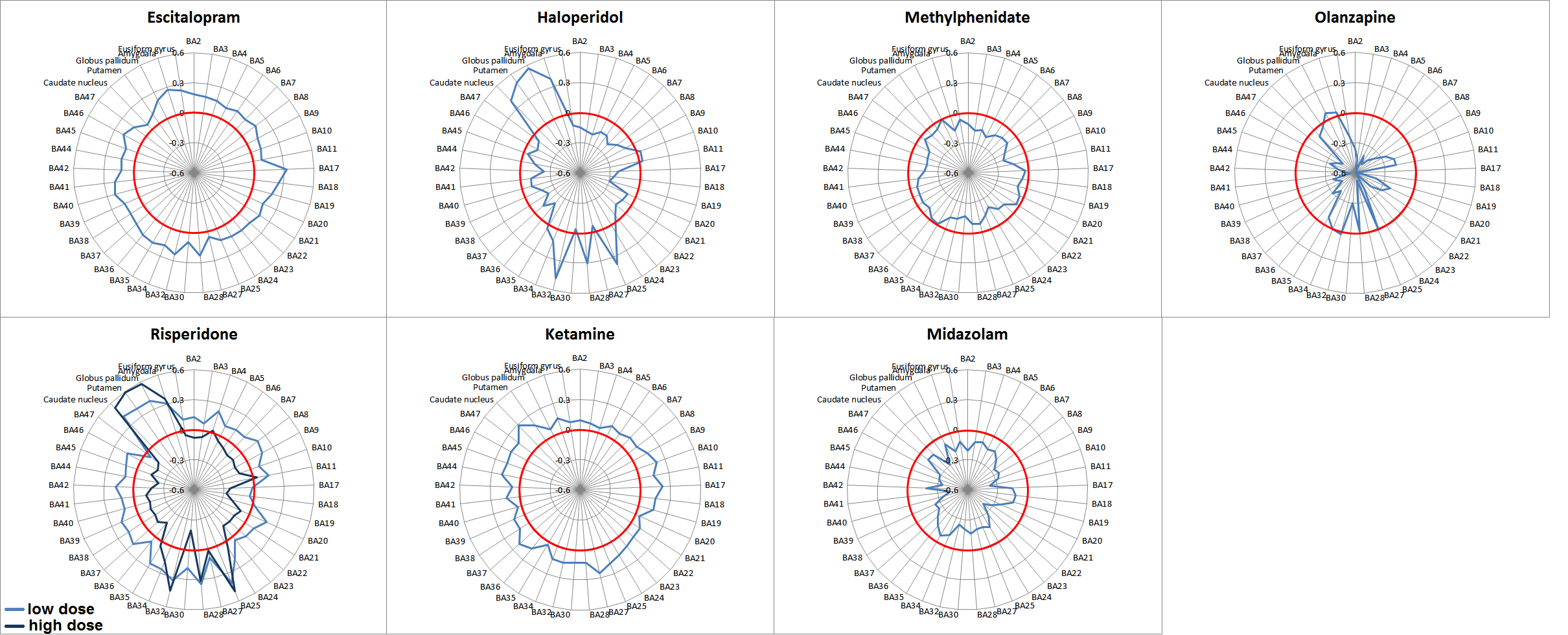
**

Effect sizes (blue lines) are displayed as spider plots for respective drugs and regions. Red line indicates zero change, BA – Brodmann area

**Figure S5** Results of Spearman correlations between receptor densities and CBF changes

**
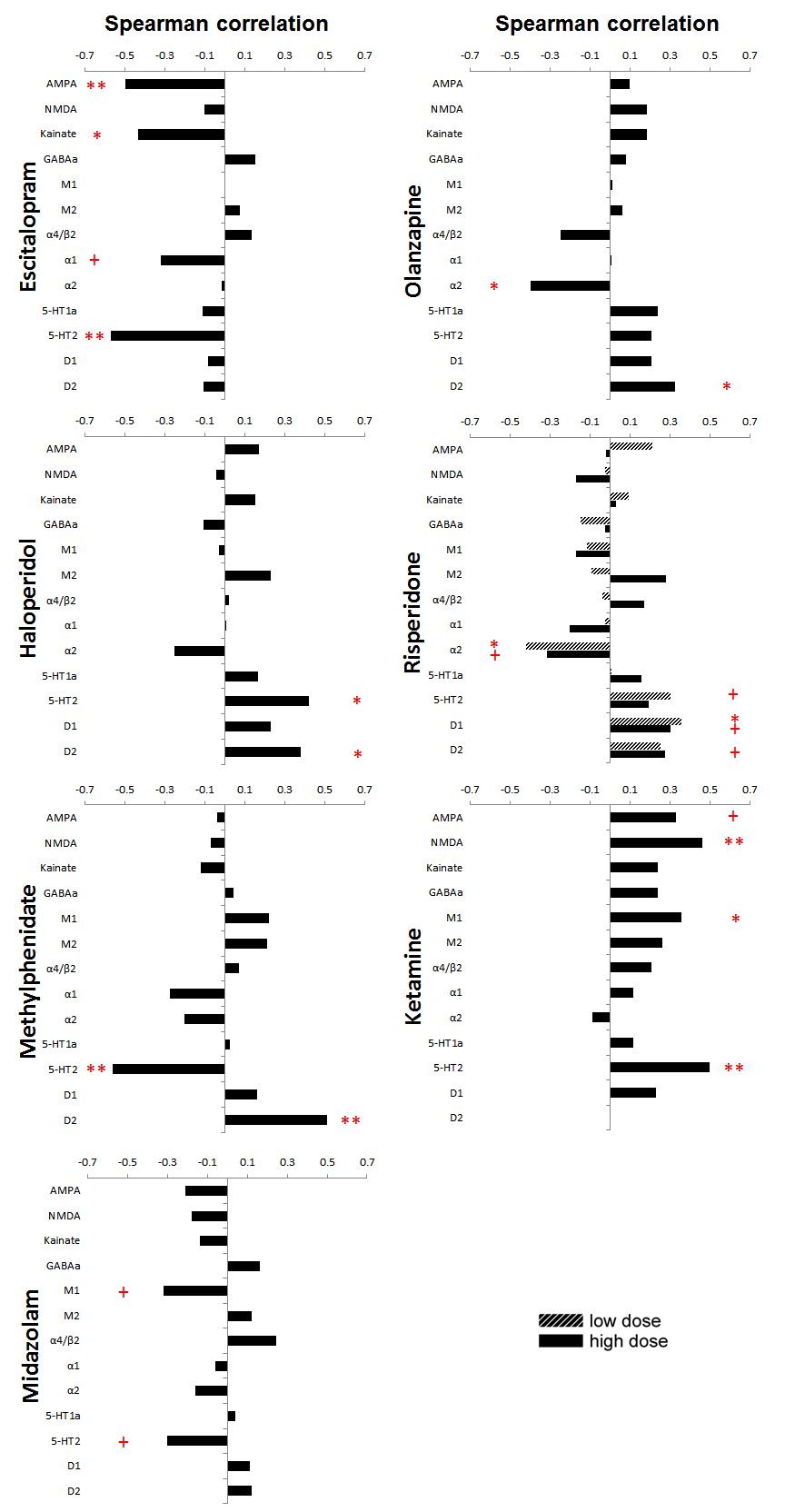
**

** Significant Bonferroni corrected association, * Significant uncorrected (p<.05) association, + Marginally significant association

**Figure S6** Representative single subject CBF data for the different studies in Montreal Neurological Institute space


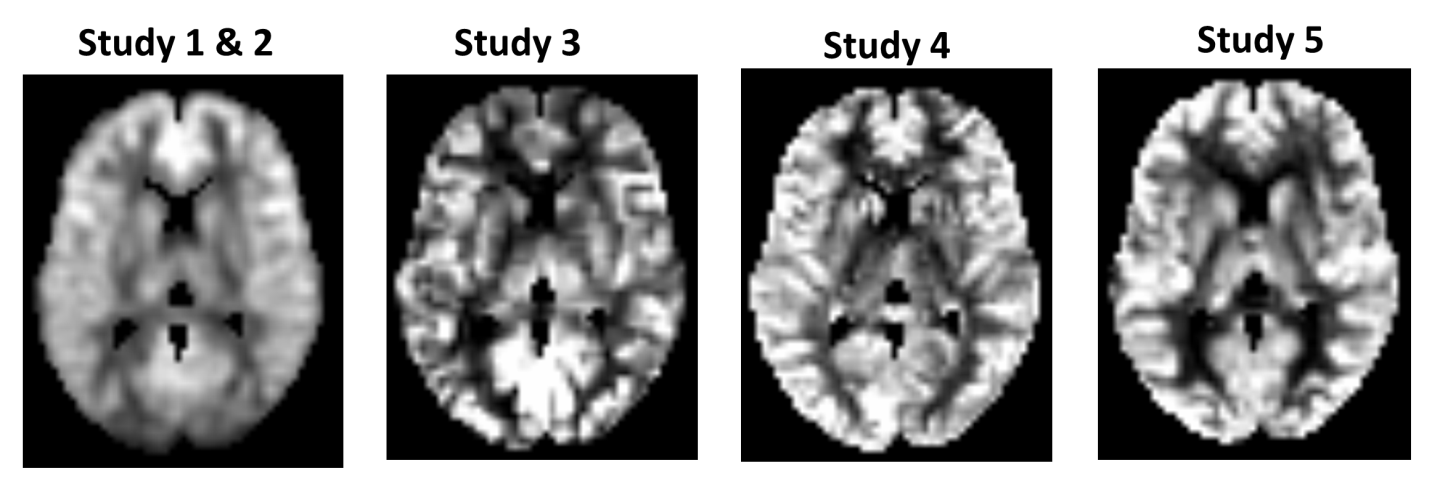


**Table S1** Overview of sequence details

| **Study site** | **UCL** | **UCSD** | **UA** | **UG** |
| --- | --- | --- | --- | --- |
| **Study IDs** | 1,2 | 3 | 4 | 5 |
| **Scanner type** | GE MR750 3.0T | GE Signa EXCITE 3.0T | Siemens Magnetom Skyra 3.0T | Philips Intera 3.0T |
| **ASL type** | PCASL | FAIR QUIPSS II | PCASL | PCASL |
| **Sequence details** | TR=4.968s,  TE=11.1ms,  LDur=1.5s, LDel=1.5s | TI1=600ms, TI2=1600ms, TR=2.5s, TE=3ms | TR=4.5s, TE=15.6ms, LDur=1.8s, LDel=1.8s | TR=4s, TE=14ms, LDur=1.65s, LDel=1.6s |
| **Resolution (in mm)** | 2x2x3 | 3.4x3.4x5 | 3x3x3 | 3x3x7 |

ASL – arterial spin labeling, KCL – King’s College London, LDur – labeling duration, LDel – labeling delay, PCASL – pseudo-continuous ASL, TR – repetition time, TE – echo time, TI – inversion time, UCSD – University of California San Diego, UA – University of Auckland, UG – University of Groningen

**Table S2** Receptor density maps

| Region | AMPA | NMDA | Kainate | GABAa | M1 | M2 | Nicotinic α4β2 | α1 | α2 | 5-HT 1a | 5-HT 2 | D1 | D2 |
| --- | --- | --- | --- | --- | --- | --- | --- | --- | --- | --- | --- | --- | --- |
| BA2 | 1 | 2 | 2 | 2 | 2 | 2 | 1 | 2 | 2 | 2 | 2 | 1 | 1 |
| BA3 | 1 | 1 | 2 | 2 | 1 | 3 | 2 | 1 | 3 | 1 | 2 | 1 | 1 |
| BA4 | 1 | 1 | 1 | 1 | 1 | 1 | 2 | 1 | 1 | 1 | 1 | 1 | 1 |
| BA5 | 1 | 2 | 2 | 2 | 3 | 2 | 1 | 2 | 2 | 2 | 2 | 1 | 1 |
| BA6 | 1 | 2 | 2 | 2 | 1 | 2 | 2 | 2 | 2 | 2 |  | 1 | 1 |
| BA7 | 2 | 2 | 3 | 2 | 2 | 3 | 1 | 3 | 3 | 2 | 2 | 2 | 1 |
| BA8 | 2 | 2 | 2 | 2 | 2 | 2 | 1 | 2 |  |  |  | 1 | 1 |
| BA9 | 2 | 3 | 3 | 2 | 2 | 1 | 1 | 3 | 2 | 2 | 3 | 1 | 1 |
| BA10 |  |  |  | 2 | 2 | 2 |  |  |  | 2 | 3 | 1 | 1 |
| BA11 |  |  | 2 | 2 |  |  | 1 | 1 |  | 2 | 3 | 1 | 1 |
| BA17 | 1 | 2 | 1 | 3 | 3 | 3 | 2 | 1 | 3 | 1 | 2 | 2 | 1 |
| BA18 | 1 | 2 | 2 | 2 | 2 | 2 | 2 | 2 | 2 | 2 | 1 | 1 | 1 |
| BA19 | 1 | 2 | 2 | 2 | 2 | 2 | 2 | 2 | 2 | 2 | 1 | 1 | 1 |
| BA20 |  | 2 | 2 | 2 | 2 | 2 | 1 | 2 | 2 | 2 | 1 | 1 | 1.5 |
| BA21 | 2 | 2 | 3 | 2 | 3 | 2 | 1 | 2 | 2 | 2 | 1 | 1 | 1.5 |
| BA22 |  | 2 | 3 | 2 | 2 | 2 | 1 | 2 | 2 | 2 | 1 | 1 | 1.5 |
| BA23 | 2 |  | 2 | 2 | 2 | 2 | 2 | 3 | 2 | 2 | 2 | 1 |  |
| BA24 | 3 | 1 | 3 | 2 | 1 | 2 | 1 | 3 | 3 | 2 | 2.5 | 2 | 1 |
| BA25 | 3 | 3 | 2 | 2 | 1 | 2 | 1 | 3 | 2 | 3 | 3 | 2 | 1 |
| BA27 | 1 | 2 | 2 |  | 2 | 3 | 2 |  |  | 3 | 3 | 1 | 1 |
| BA28 | 1 | 2 | 2 |  | 2 | 3 | 2 |  |  | 3 | 3 | 1 | 1 |
| BA30 | 3 | 2 | 2 | 2 | 2 | 3 | 1 | 2 | 2 | 2 | 1 | 1 | 2 |
| BA32 | 2 | 1 | 2 | 2 | 1 | 2 | 1 | 3 | 2 | 2 | 3 | 1 | 1 |
| BA34 | 1 | 2 | 2 |  | 2 | 3 | 2 |  |  | 3 | 3 | 1 | 1 |
| BA35 | 1 | 2 | 2 |  | 2 | 3 | 2 |  |  | 3 | 3 | 1 | 1 |
| BA36 | 1 | 2 | 3 | 2 | 2 | 2 | 1 | 2 | 2 |  | 1 | 1 | 1.5 |
| BA37 |  | 2 | 2 | 2 |  | 2 | 1 | 2 | 2 | 2 | 1 | 1 | 1 |
| BA38 |  | 2 |  |  |  | 2 |  |  | 2 |  |  | 1 | 1.5 |
| BA39 | 2 | 2 | 2 | 2 | 2 | 1.5 | 1 | 2.5 | 1 | 2 | 1 | 2 | 1 |
| BA40 | 3 | 2 | 2 | 2 | 2 | 2 | 1 | 2 | 2 | 2 | 2 | 2 | 1 |
| BA41 | 1 | 1 | 1 | 1 | 2 | 2 | 1 | 1 | 3 | 1 | 2 | 1 | 1 |
| BA42 | 1 | 1 | 1 | 1 | 2 | 3 | 2 | 1 | 3 | 1 | 2 | 1 | 1 |
| BA44 | 2 | 2 | 2 | 2 | 2 | 1 | 1 | 2 | 3 | 1 | 3 | 1 | 1 |
| BA45 | 1 | 3 | 2 | 2 | 2 | 2 | 1 | 2 | 2 | 1 | 3 | 1 | 1 |
| BA46 | 2 | 2 | 3 | 1 | 2 | 2 | 1 | 3 | 3 | 2 | 3 | 2 | 1 |
| BA47 | 3 | 3 | 3 | 2 | 3 | 1 | 1 | 1 | 1 | 1 | 2 | 1 | 1 |
| Caudate nucleus | 3 | 2 | 3 | 2 | 3 | 3 | 2 | 1 | 1 | 1 | 3 | 3 | 3 |
| Putamen | 3 | 2 | 3 | 2 | 3 | 3 | 2 | 1 | 1 | 1 | 3 | 3 | 3 |
| Globus pallidum | 1 | 1 | 1 | 1 | 1 | 1 | 1 | 1 | 1 | 1 | 1 | 2 | 2 |
| Amygdala | 1 | 2 | 1 | 3 | 2 | 2 | 1 | 2 | 1 | 1 | 1 | 2 | 1 |
| Fusiform gyrus | 2 | 2 | 2 | 3 | 1 | 3 | 1 | 2 | 2 | 2 | 1 | 2 | 1 |

Receptor density maps extracted from Palamero-Gallagher et al. (Palomero-Gallagher et al., 2015). 1 = low density, 2 = intermediate density, 3 = high density, BA – Brodmann area

**Table S3** Receptor affinity profiles for olanzapine, risperidone and haloperidol

| **Receptor type** | **Affinity (Ki in nM) profile for:** | | |
| --- | --- | --- | --- |
|  | **Olanzapine** | **Risperidone** | **Haloperidol** |
| **AMPA** | >10000 | >10000 | >10000 |
| **NMDA** | >10000 | >10000 | >10000 |
| **Kainate** | >10000 | >10000 | >10000 |
| **GABAa** | >10000 | >10000 | >10000 |
| **m1** | 1.9 | >10000 | 1475 |
| **m2** | 18 | >10000 | 1200 |
| **Niconitic α2β4** | >10000 | >10000 | >10000 |
| **α1** | 19 | 2 | 46 |
| **α2** | 230 | 3 | 360 |
| **5-HT 1a** | 1000 | 490 | 7930 |
| **5-HT 2** | 7.5 | 13.3 | 1581.5 |
| **D1** | 31 | 75 | 25 |
| **D2** | 11 | 3 | 1 |

Data were extracted from Bymaster et al. (Bymaster et al., 1996), Ki – inhibitory constant reflecting binding affinity

**References**

Alsop DC, Detre JA, Golay X, Günther M, Hendrikse J, Hernandez‐Garcia L, Lu H, MacIntosh BJ, Parkes LM, Smits M (2015) Recommended implementation of arterial spin‐labeled perfusion MRI for clinical applications: A consensus of the ISMRM perfusion study group and the European consortium for ASL in dementia. Magn Reson Med 73:102–116.

Bymaster FP, Calligaro DO, Falcone JF, Marsh RD, Moore NA, Tye NC, Seeman P, Wong DT (1996) Radioreceptor binding profile of the atypical antipsychotic olanzapine. Neuropsychopharmacology 14:87–96.

Dai W, Garcia D, de Bazelaire C, Alsop DC (2008) Continuous flow-driven inversion for arterial spin labeling using pulsed radio frequency and gradient fields. Magn Reson Med 60:1488–1497.

Friston KJ, Holmes AP, Worsley KJ, Poline J-P, Frith CD, Frackowiak RS (1994) Statistical parametric maps in functional imaging: a general linear approach. Hum Brain Mapp 2:189–210.

Kroeze WK, Hufeisen SJ, Popadak BA, Renock SM, Steinberg S, Ernsberger P, Jayathilake K, Meltzer HY, Roth BL (2003) H1-histamine receptor affinity predicts short-term weight gain for typical and atypical antipsychotic drugs. Neuropsychopharmacology 28:519–526.

Myers JF, Rosso L, Watson BJ, Wilson SJ, Kalk NJ, Clementi N, Brooks DJ, Nutt DJ, Turkheimer FE, Lingford-Hughes AR (2012) Characterisation of the contribution of the GABA-benzodiazepine α1 receptor subtype to [11C] Ro15-4513 PET images. J Cereb Blood Flow Metab 32:731–744.

Palomero-Gallagher N, Amunts K, Zilles K (2015) Transmitter Receptor Distribution in the Human Brain. In: Brain Mapping: An Encyclopedic Reference, pp 261–275. Elsevier.

Rorden C, Brett M (2000) Stereotaxic display of brain lesions. Behav Neurol 12:191–200.
